# A Method to Encapsulate Small Organic Molecules in Calcium Phosphate Nanoparticles Based on the Supramolecular Chemistry of Cyclodextrin

**DOI:** 10.3390/mi8100291

**Published:** 2017-09-27

**Authors:** Zhongming Zhu, Feng Li, Fei Zhong, Kang Zhai, Wei Tao, Gengyun Sun

**Affiliations:** 1 Pulmonary Department, First Affiliated Hospital of Anhui Medical University, Hefei 230022, China; zhuzhongming@ahmu.edu.cn; 2 Department of respiration, Shanghai Public Health Clinical Center, Fudan University, Shanghai 201508, China; lifeng@shaphc.org; 3 Department of Oncology, Fuyang Hospital of Anhui Medical University, Fuyang 236000, China; 4 School of Biological and Medical Engineering, Hefei University of Technology, Hefei 230009, China; zkhj19931015@gmail.com (K.Z.); taow@hfut.edu.cn (W.T.)

**Keywords:** calcium phosphate nanoparticles, supramolecular chemistry, cyclodextrin, small organic drug delivery

## Abstract

Calcium phosphate nanoparticles (CPNPs) encapsulating small organic molecules, such as imaging agents and drugs, are considered to be ideal devices for cancer diagnosis or therapy. However, it is generally difficult to encapsulate small organic molecules in CPNPs because of the lack of solubility in water or binding affinity to calcium phosphate. To solve these issues, we utilized the carboxymethyl β-cyclodextrin (CM-β-CD) to increase the solubility and binding affinity to small organic molecules for the encapsulation into CPNPs in this work. The results indicated that the model molecules, hydrophilic rhodamine B (RB) and hydrophobic docetaxel (Dtxl), are successfully encapsulated into CPNPs with the assistance of CM-β-CD. We also demonstrated the CPNPs could be remarkably internalized into A549 cells, resulting in the efficient inhibition of tumor cells’ growth.

## 1. Introduction

An ideal carrier for delivering bioactive agents should have several features, including biocompatibility, safety, and controlled drug release [1,2,3]. Calcium phosphate (CaP), the major component of human bones and teeth, is highly biocompatible, and relatively insoluble at a pH above 7.4, but dissolves into calcium and phosphate ions at a pH below 6. When bioactive agents are encapsulated in nano-sized Calcium phosphates (CaPs), they will be protected from the outer environment and prevented from undesired release to normal tissues or cells during circulation (~pH 7.4); after accumulating in solid tumor tissue and internalizing into tumor cells, these agents are finally released in endosomes (pH ~ 5.4) or lysosomes (pH ~ 4.5). Therefore, calcium phosphate nanoparticles (CPNPs) have been considered one of the most promising delivery devices for cancer diagnosis or therapy [4,5,6].

Various nuclei acids [7,8,9], proteins [10,11,12], or polysaccharides [13,14,15] have been encapsulated into CPNPs based on a precipitation reaction of the CaPs in water with the presence of these biomacromolecules. However, it is difficult to encapsulate small hydrophobic organic molecules in CPNP because the preparation of CaP is performed in water, while the hydrophobic organic molecule was insoluble during the preparation [16]. Additionally, the binding affinity of these molecules to CaP should be strong enough to ensure the efficient encapsulation [17,18,19]. Two strategies have been developed to encapsulate small organic molecules into CPNPs. The first strategy, pioneered by Adair and co-workers, is preparing the CPNPs by a double reverse emulsion procedure using disodium silicate as a nucleation agent [20,21,22]. However, this method is limited by the solubility of the molecules and the addition of organic solvents [4,16]. The other strategy is using assistant nanoparticles for the simultaneous functions of encapsulating and binding, such as polymeric self-assemblies [23,24,25,26,27,28], liposomes [29], mesoporous silica [30], or even viruses [31] and cells [32]. However, the preparation of these assistant nanoparticles often requires sophisticated skills.

**Figure 1:**
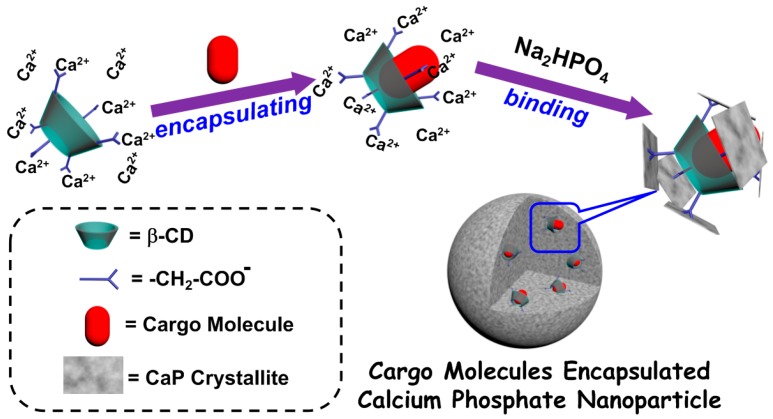
Illustration of the functions of CM-β-CD in preparation of cargo molecules encapsulated calcium phosphate nanoparticles.

Cyclodextrins (CDs) are cyclic oligosaccharides with the ability to form complexes with a wide spectrum of drug molecules via noncovalent interactions in their hydrophobic cavities [33,34], and the hydroxy groups around their rims can be modified to endow strong binding ability to CaP [35,36]. CDs and their derivatives have been widely used to improve the performance of bulk CaPs [37] or to decorate the surfaces of CPNPs [38]. Recently, Raj and co-workers synthesized CPNPs using β-CD as a medium to conjugate rhodamine isocyanide for H_2_O_2_ detection [39]. Inspired by these studies, we proposed a facile method to encapsulate small organic molecules into CPNPs with the assistant of CDs (Figure 1). We prepared carboxymethyl β-cyclodextrin (CM-β-CD) to afford the functions of encapsulating cargo molecules and binding to CaP crystallites simultaneously. When CM-β-CD forms noncovalent complexes with small organic molecules, it not only improves their solubility but also forms CaPs occurring around the host–guest pairs through interactions between carboxy groups and CaPs. We chose rhodamine B (RB) and docetaxel (Dtxl) as model molecules to represent hydrophilic and hydrophobic molecules, respectively. The results indicated that RBs and Dtxls could be efficiently encapsulated into CPNPs with the assistance of CM-β-CD.

## 2. Materials and Methods

### 2.1. Materials and Characterization

Calcium chloride (CaCl_2_), disodium hydrogen phosphate (Na_2_HPO_4_), β-cyclodextrin (β-CD), sodium citrate, sodium hydroxide (NaOH), concentrated hydrochloric acid (HCl, 36.5%), chloroacetic acid, and ethanol were purchased from Sinopharm Chemical Reagent Co., Ltd. (Shanghai, China). Rhodamine B (RB) and docetaxel (Dtxl) were purchased from Shanghai Aladin Co., Ltd. (Shanghai, China). Dimethyl sulfoxide (DMSO) and [3-(4,5-dimethylthiazol-2-yl)-2 and 5-diphenyltetrazolium bromide] (MTT) were purchased from Sigma-Aldrich (St. Louis, MO, USA). All reagents were of analytical grade and used as received.

Dynamic Light Scattering (DLS) measurements were performed under a Malvern Instruments Zetasizer Nano series instrument (ZS90, Malvern, Worcestershire, UK) equipped with a 22 mW laser light and operating at a wavelength of 633 nm. All samples were about 1 mg/mL and measured at 25 °C with a scattering angle of 90°. Transmission electron microscopy (TEM) measurements were performed with an H-800 electron microscope (Hitachi, Tokyo, Japan). The ultraviolet-visible (UV-Vis) absorbance experiments were performed with a UV-7504 spectrophotometer (Shanghai Xinmao Ltd., Shanghai, China). High-performance liquid chromatography (HPLC) was performed on a HP1100 (Agilent, Oshawa, ON, Canada) system with an Atlantis column as stationary phase at 25 °C. A mixture of acetonitrile and water (55:45) was used as mobile phase at a flow rate of 1 mL/min. Detection was performed with a diode-array detector at a detection wavelength of 230 nm. 

### 2.2. Synthesis of RBs-Encapsulated CPNPs (RBs-en-CPNPs)

To a mixture of CM-b-CD (20 mg) and RBs (8 mg) in an aqueous solution of CaCl_2_ (20 mL, 0.04 M), Na_2_HPO_4_ in water (12 mL, 0.04 M) was added dropwise within 30 min. Subsequently, the mixture was adjusted to pH 10 by an aqueous solution of NaOH (1 M) and stirred for 1 h. Then, 30 mg of sodium citrate was added. After further stirring for 1 h, the mixture was dialyzed against (MWCO: 3500 Da) water for 24 h. Then, the mixture was centrifuged at 10,000 r/m for 15 min, and the obtained precipitate was re-dispersed in ethanol, centrifuged at 10,000 r/m for 15 min to remove the residual RBs three times. The final product was collected and dried at 40 °C under a vacuum.

To investigate if the RBs can be encapsulated into CaPs directly, we prepared CaPs in the aqueous solution of RBs. The synthesis process was as same as the one for RBs-en-CPNPs, except that CM-b-CD was absent. Moreover, we also prepared the CaPs, and then the RBs and CM-b-CD was added. The obtained formulation was used as a control. 

### 2.3. Synthesis of Dtxls-Encapsulated CPNPs ((Dtxls-en-CPNPs))

Briefly, 25 mg of CM-b-CD and 2.5 mg of Dtxls were added to an aqueous solution of CaCl_2_ (20 mL, 0.04 M). After ultrasonication for 1 h, an aqueous solution of Na_2_HPO_4_ (12 mL, 0.04 M) was added dropwise within 30 min. Then, the mixture was adjusted to pH 10 by an aqueous solution of NaOH (1 M) and stirred for 1 h. Subsequently, 30 mg of sodium citrate was added, stirred for an additional 1 h, and then transfer into dialysis bag (MWCO: 3500 Da) and dialysis against water for 24 h. Then, the mixture was centrifuged at 10,000 r/m for 15 min. The obtained precipitate was re-dispersed in ethanol and then centrifuged at 10,000 r/m for 15 min to remove residual Dtxls. The final product was collected and dried at 40 °C under vacuum. Moreover, we also prepared the control formulation; the Dtxls was added the previously formed CaPs. Blank CPNPs were prepared by the similar synthesis process, except that Dtxls were absent.

### 2.4. Calculation of Loading Content and Loading Efficiency

The loading efficiency (LE) and loading content (LC) were calculated according to the equations:

(1) $$\mathrm{LE}\%=\frac{\mathrm{Weight}\;\mathrm{of}\;\mathrm{drug}\;\mathrm{found}\;\mathrm{loaded}}{\mathrm{Weight}\;\mathrm{of}\;\mathrm{drug}\;\mathrm{input}}\times 100\%$$

(2) $$\mathrm{LC}\%=\frac{\mathrm{Weight}\;\mathrm{of}\;\mathrm{drug}\;\mathrm{found}\;\mathrm{loaded}}{\mathrm{Weight}\;\mathrm{of}\;\mathrm{drug}\;\mathrm{loaded}\;\mathrm{nanoparticles}}\times 100\%.$$

Briefly, 12 mg of RBs-en-CPNPs were added to 10 mL of HCl solution (pH 1). The mixture was treated by ultrasonication for at least 30 min, then lyophilized to remove water. After that, 10 mL of PBS (pH 5.4) was added and then measured by UV spectrophotometry at a wavelength of 555.7 nm. The quantity was calculated according to the standard curve of UV absorbance intensities to concentrations of RBs.

For Dtxls-en-CPNPs, 5 mg of Dtxls-encapsulated CPNPs were added to 5 mL of HCl solution (pH: 1). The mixture was treated by ultrasonication for 30 min, then lyophilized to remove water. After that, 10 mL of methanol was added. The mixture was treated by a vortex mixer for 10 min, centrifuged for 10 min at 10,000 rmp, and then 20 µL of supernatant was measured by HPLC with acetonitrile/water (55:45, *v*/*v*) as eluent. 

### 2.5. Cargo Release

Forty milligrams of cargo molecule encapsulated CPNPs were added into 20 mL PBS solution (pH 5.4 or 7.4), then the mixture was transferred to a dialysis bag (MWCO: 3500 Da). After the dialysis bag was immersed in 200 mL of corresponding PBS solution, the release was performed in a thermostatic shaker (37 °C, 150 rpm). At predetermined intervals, 2.0 mL buffer solution outside the dialysis bag was extracted and it was replaced by an equal volume of fresh media to keep the thermostatic shaker. The amount of released cargo molecules was analyzed by UV absorbance (RBs) or HPLC (Dtxl).

### 2.6. Cell Culture

The human lung cancer cell line A549 (ATCC) (Shanghai Fumengjiyin Biotechnology Co., Ltd., Shanghai, China) were cultured in DMEM (Gibco, Carlsbad, CA, USA) with 10% fetal bovine serum (Gibco) at 37 °C with 5% CO_2_ humidified atmosphere. 

### 2.7. Flow Cytometry

A549 cells were seeded into six-well plates at 1 × 10^5^ cells per well in 0.5 mL of complete DMEM medium and cultured at 37 °C in a 5% CO_2_ humidified atmosphere for 24 h. The medium was replaced with DMEM medium containing RBs-en-CPNPs at a RBs dose of 20 mM. The cells were incubated at 37 °C for 1 h, 2 h, or 4 h, and then the cells were rinsed, trypsinized, and subjected to flow cytometric analysis using a BD FACSCalibur flow cytometer (BD Bioscience, Bedford, MA, USA). 

### 2.8. Confocal Microscopy

A549 cells were incubated with RBs-en-CPNPs at a RBs dose of 20 mM for 1 h, 2 h, or 4 h, and then washed twice with PBS, and fixed with 4% formaldehyde for 15 min at room temperature. The cell cytoskeleton F-actin and cell nuclei were counterstained with Alexa Fluor 488 and DAPI respectively. Coverslips were mounted on glass microscope slides with a drop of anti-fade mounting media (Sigma-Aldrich) to reduce fluorescence photobleaching. The cellular uptake of RBs-en-CPNPs was visualized by a confocal laser scanning microscope (LSM 710, Carl Zeiss Inc., Jena, Germany). 

### 2.9. Cytotoxicity Assay

The cytotoxicity of Dtxl-en-CPNPs was measured by MTT test. Typically, A549 cells were cultured in 96-well plates at a density of 1.0 × 10^4^ cells per well. Subsequently, serial dilutions of CPNPs were added. After incubation for 48 h, the samples were treated with MTT for 4 h. The formed formazan crystals were then dissolved in DMSO and absorbance was measured at 570 nm on a microplate reader (Model 680; Bio-Rad, Hercules, CA, USA).

## 3. Results and Discussion

### 3.1. Synthesis of Small Organic Molecule Encapsulated CPNPs

**Figure 2:**
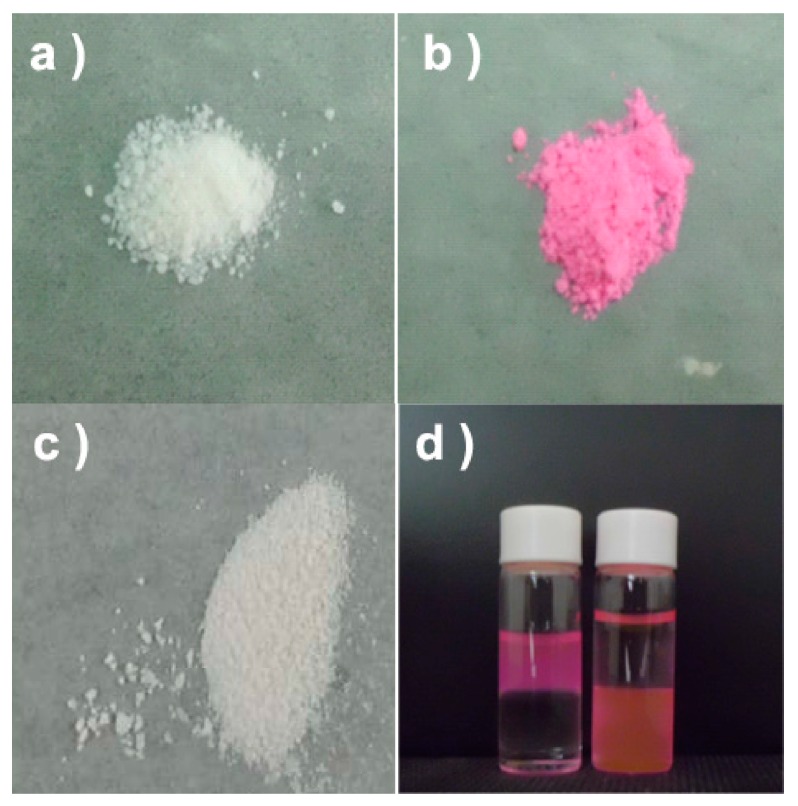
(**a**–**c**) The photographs of the CaPs synthesized in the solutions of RBs (**a**) without, (**b**) with the CM-b-CD and (**c**) by adding the solution of RBs and CM-b-CD after the CaPs formed; (**d**) the imagine of the RBs encapsulated CaPs in the water (upper layer)/chloroform (bottom layer) system before (left bottle) and after (right bottle) concentrated HCl was added.

In this work, the CPNPs were firstly synthesized by the simple precipitation reaction of CaCl_2_ and Na_2_HPO_4_ in water with RBs or Dtxls, then stabilized by sodium citrate, and finally laundered by dialysis against water and subsequent washing with ethanol. However, only withe powder was obtained after laundering process (Figure 2a). The loading content (LC) and loading efficiency (LE) of RB were ca. 0.0140% and 1.28%, respectively, indicating that RB could barely be encapsulated directly into CaPs.

CD or CD derivatives are able to accommodate the lactone group of RB's lactone form or the diethylaminophenyl group of its carboxylate ion form in their cavities [40]. Thus, the CM-b-CD was synthesized by reaction of b-CD and chloroacetic acid under alkaline condition, and ^1^H NMR indicated that the carboxymethyl groups were grafted on the rim of b-CD with the substitution number about 6 (ESI, Figure S1). Subsequently, the formation of supramolecular complex between RB and CM-b-CD was investigated. The result of 2D NOESY spectroscopy revealed that RB and CM-b-CD were able to form complex in the condition for preparing CaPs with the latter mode (ESI, Figure S2). When CaPs were synthesized in the aqueous solution of RBs in the presence of CM-b-CD, pink powder was obtained after laundering process (Figure 2b). UV-vis absorbance experiments further indicated that absorbance of RB in PBS was not affected by addition of CM-b-CD (ESI, Figure S3), neither in pH 7.4 nor pH 5.4. Therefore, the LC and LE of RB, which were calculated according to UV-vis absorbance, were 0.82% and 32.72%, respectively. Changing the feeding order can be used to estimate if the interested materials are encapsulated [41], therefore, we synthesized another control sample by adding the mixture of RBs and CM-b-CD after the formation of CaPs. The product was a white powder (Figure 2c), and the LC and LE of RBs 0.0037% and 0.012%, respectively. This result indicated that CM-b-CD would not assist RBs anchoring on the surface of CaPs. Furthermore, to corroborate the encapsulated feature of the RBs and the pH-sensitive property of the CaPs, extraction experiments were operated. As shown in Figure 2d, the RBs, carried by the pink powder, were stable in water phase and extracted into chloroform (Figure 2d, left bottle). However, after being added several drops of concentrated HCl, the upper aqueous phase became transparent, and the RBs transported to the chloroform phase almost entirely (Figure 2d, right bottle). Because RBs cannot bind to the surface of CaPs tightly, the extraction experiments proved that the RBs were incorporated inside CaPs and acidic conditions would make them exposed.

Dtxl cannot be encapsulated in CPNP directly because of its poor solubility in water. When CM-b-CD was added into the suspension of Dtxls in CaCl_2_ solution, the formation of a transparent solution indicated the supramolecular complexes formed, which were further verified by 2D NOESY experiment (ESI, Figure S4). After an analogous synthesis procedure to the one for RBs encapsulated CaPs, the Dtxls-loaded CaPs were obtained. The quantity of Dtxls-loaded CaPs was characterized with HPLC [42]. The LC and LE were 6.24% and 36.25%, respectively. However, when the solution of Dtxls and CM-b-CD was added after the formation of CaPs, the LC and LE were only 0.11 wt % and 3.34 wt %, respectively, implying that the Dtxls must added during the formation of CaPs.

**Figure 3:**
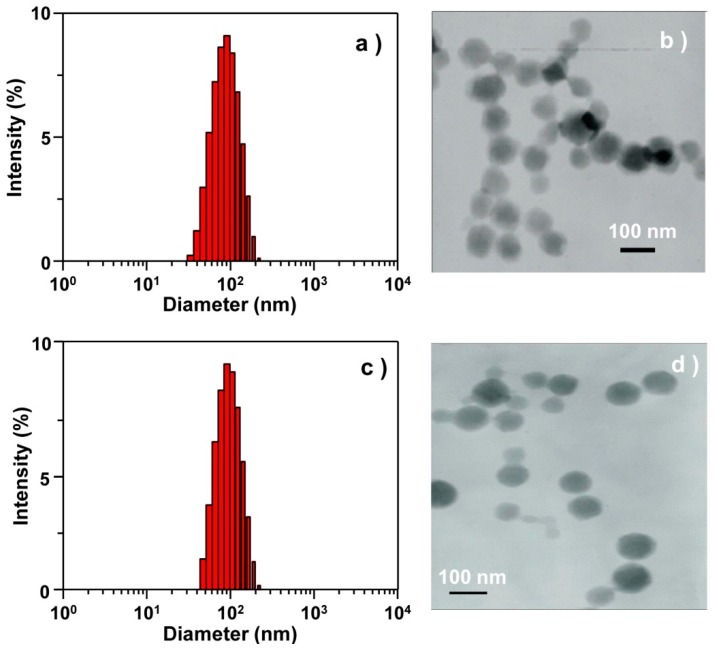
(**a**,**c**) Intensity distributions of the sizes of (**a**) RB and (**c**) Dtxls encapsulated CaPs; (**b**,**d**) TEM images of (**b**) RB and (**d**) Dtxls-encapsulated CaPs.

To investigate the size of the cargo molecule-encapsulated CaPs, DLS and TEM were used. The diameters of the RBs-en-CaPs observed by DLS were about 100 nm (Figure 3a), which coincided with the observation from TEM (Figure 3b). On the contrary, the diameters of the Dtxls-en-CaPs increased to 200 nm by DLS (Figure 3c), which was further confirmed by TEM (Figure 3d). These results for the RBs-en-CPNPs and Dtxls-en-CPNPs were successfully synthesized with the assistance of CM-b-CD. Additionally, the stability of RBs-en-CPNPs and Dtxls-en-CPNPs was also detected [43]. As shown in Figure S5, the sizes showed almost no increase in PBS containing 10% FBS, exhibiting excellent stability.

### 3.2. In Vitro Release of the Cargo Molecules from CPNPs

**Figure 4:**
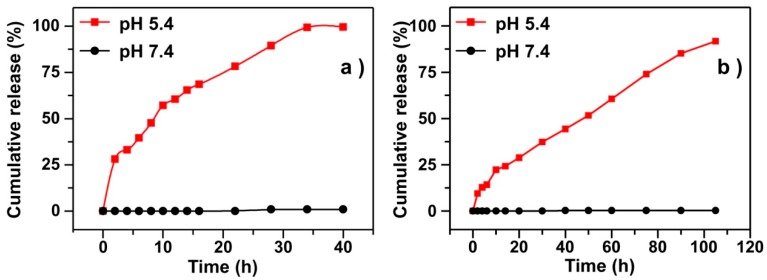
Release profiles of (**a**) RBs and (**b**) Dtxls from the corresponding CPNPs at pH 7.4 and 5.4.

CPNPs are a kind of smart vehicle because of its pH dependent solubility. To demonstrate it, the release profiles of RBs-en-CaPs and Dtxls-en-CaPs at pH 7.4 and 5.4 were analyzed. For the RBs-en-CaPs (Figure 4a), there were nearly no release of RBs at pH 7.4, while the RBs were almost entirely released within 40 h at pH 5.4, implying that the CPNPs was able to prevent the release of the cargoes during circulation and then release them after the nanoparticles enter into an acidic endosome/lysosome. For Dtxls-en-CaPs (Figure 4b), a similar release profile was observed. However, to achieve complete release, the time for Dtxls-en-CaPs was about three times that of RBs-en-CaPs. We supposed this phenomenon might be caused by two reasons: one is that the dimeter of Dtxls-en-CPNPs was about double that of RBs-en-CPNPs, and the larger superficial area of the RBs-en-CPNPs meant RBs were exposed more quickly under acidic conditions; the other is that the diffusion of the Dtxls in water might be slower because of their hydrophobic nature.

### 3.3. Cellular Uptake of the CPNPs

**Figure 5:**
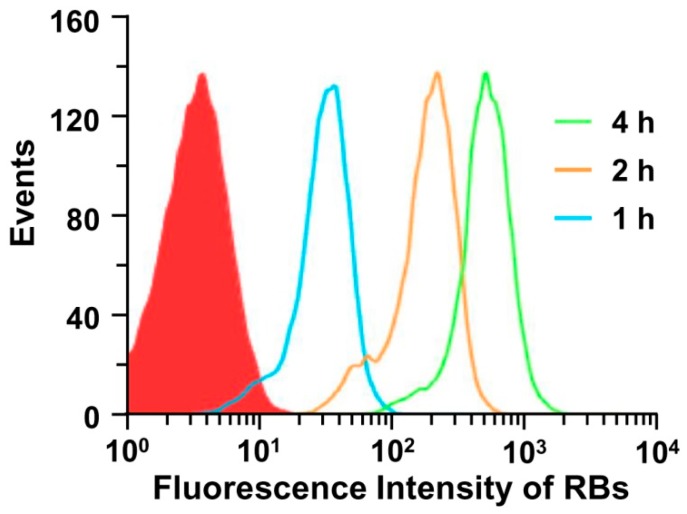
Flow cytometry analyses of the cellular uptake of RBs-en-CPNPs.

As reported, the nanoparticles can be internalized into tumor cells via the endocytosis pathway [44]. To observe the cell internalization of the CPNPs, RBs-en-CPNPs were used for flow cytometry analyses because of the fluorescent property of RB. A549 cells were incubated with RBs-en-CPNPs at a RBs dose of 20 mM. After incubation for different times, the intracellular RBs’ fluorescence was determined by flow cytometric analysis. As shown in Figure 5, the strong intracellular fluorescence of A549 cells was detected, indicating that the CPNPs were internalized by the cells. Meanwhile, the fluorescence intensity of the cells increased with the extension of culturing time from 1 h to 4 h, indicating continual cellular uptake.

**Figure 6:**
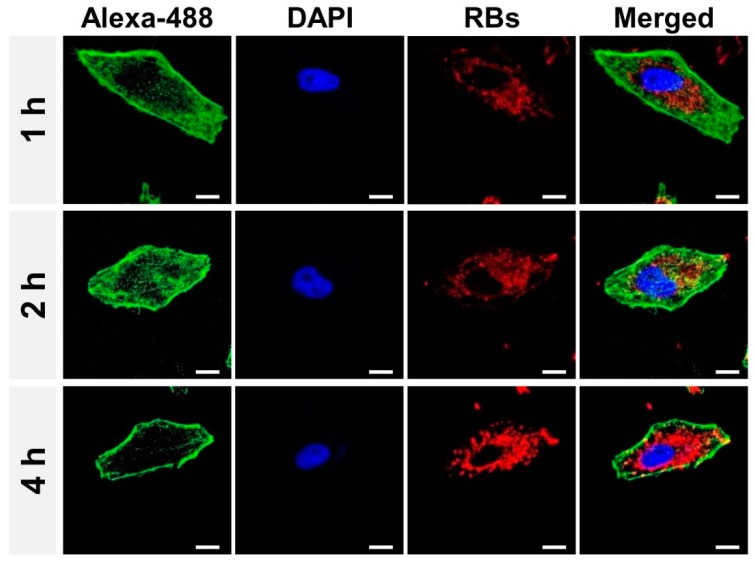
Confocal laser scanning microscopy observation of A549 cells incubated with RBs-en-CPNPs. The red fluorescence represented the intracellular RBs signal. Cell nuclei and the cytoskeleton were stained with DAPI (Blue) and Alexa Fluor 488 phalloidin (Green), respectively. Scale bar = 10 μm.

Furthermore, confocal laser scanning microscopy (CLSM) analysis was further used to evaluate the cellular uptake of the CPNPs. A549 cells were incubated with RBs-en-CPNPs at an RBs dose of 20 mM. After incubation for different times, the cytoskeleton F-actin and the cell nuclei were counter-stained with Alexa Fluor 488 and DAPI, respectively. As shown in Figure 6, the red color around the cell nuclei was clearly observed, which further confirmed the efficient cellular uptake of RBs-en-CPNPs. Additionally, it was demonstrated that the fluorescence intensity of cells incubated with RBs-en-CPNPs increased with the extension of culturing time, which was consistent with the results of flow cytometry analyses.

### 3.4. Cell Cytotoxicity

**Figure 7:**
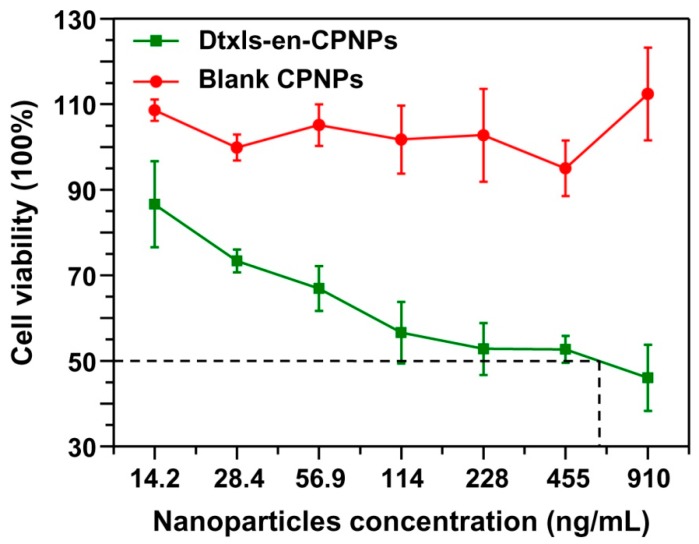
In vitro cytotoxicity profile of blank CPNPs and Dtxls-en-CPNPs against A549 cells.

As an anticancer drug, Dtxl can induce apoptosis by binding tubulin to promote polymerization and prevent the depolymerization of microtubules causing mitotic arrest [45]. To evaluate the anticancer efficacy of the Dtxls-en-CPNPs, an MTT test was performed. Additionally, blank CPNPs were used as a control. As shown in Figure 7, blank CPNPs showed almost no cytotoxicity to A549 cells (red line), indicating their good biocompatibility. However, the Dtxls-en-CPNPs greatly increased the death rate of A549 cells with the IC_50_ at about 0.037 mM, exhibiting clear activity against tumor cells.

## 4. Conclusions

In summary, we have proposed a facile method to prepare small organic molecules encapsulated CPNPs based on the supramolecular chemistry of cyclodextrins. CM-β-CD was used to afford the functions of improving the solubility of cargo molecules and binding affinity to CaP. Two model molecules, a hydrophilic dye (RB) with a lack of binding affinity to CaP and a hydrophobic anticancer drug (Dtxl) unable to dissolve in water, were able to be encapsulated in CPNPs efficiently with the assistance of CM-β-CD. Furthermore, the functional studies demonstrated the potential applications of the CPNPs as bioimaging or anticancer devices. Encouraged by in vitro results, we will carry out in vivo animal experiments to further study the biodistribution and anticancer efficacy of the CPNPs.

## Supplementary Materials

The following are available online at www.mdpi.com/2072-666X/8/10/291/s1, Figure S1: ^1^H NMR of CM-β-CD, Figure S2: (A) 2D NOESY spectrum of CM-β-CD (8.4 × 10^-4^ M) and RB (6.7 × 10^-4^ M) in CaCl_2_ solution (0.04 M) of D_2_O and (B) the illustration of the interaction mode between CM-β-CD and RB, Figure S3: UV-Vis absorbance spectra of RB with the addition of CM-β-CD in PBS solution at pH (A) 7.4 and (B) 5.4, Figure S4: 2D NOESY spectrum of CM-β-CD (1.5 × 10^-4^ M) and RB (8.4 × 10^-4^ M) in CaCl_2_ solution (0.04 M) in D_2_O, Figure S5: The size change of RBs-en-CPNPs Dtxls-en-CPNPs following incubation with PBS containing 10% FBS.
