# Supplementary material for: A Method to Encapsulate Small Organic Molecules in Calcium Phosphate Nanoparticles Based on the Supramolecular Chemistry of Cyclodextrin
Source: Micromachines (Basel). 2017 Sep 27;8(10):291. doi: 10.3390/mi8100291 (PMC6190105; doi:10.3390/mi8100291)
Supplement: Supplementary file 1 [file micromachines-08-00291-s001.pdf]

micromachines

A Method to Encapsulate Small Organic Molecules in Calcium Phosphate Nanoparticles Based on the Supramolecular Chemistry of Cyclodextrin

Zhongming Zhu, Feng Li, Fei Zhong, Kang Zhai, Wei Tao, Gengyun Sun

1. Synthesis of the Carboxymethylated β-CD (CM-β-CD)

To a mixture of β-CD (9.1 g, 8 mmol) and sodium hydroxide (7.5 g, 187.5 mmol) in distilled water (30 mL) at 60 °C, 70ml of monochloroacetic acid aqueous solution (1 M) was added dropwise for 30 min. After stirring at 60 °C for four hours, the mixture was cooled to room temperature (25 °C) and adjusted to pH 10 with concentrated hydrochloric acid. The mixture was allowed to react for an additional 12 h at room temperature before it was neutralized by concentrated hydrochloric acid. Subsequently, the mixture was poured into excess ethanol to give a translucent suspension, and the product was collected as a white precipitate by filtration. The precipitate was redissolved in water and re-precipitated in ethanol twice and then dried at 40 °C under a vacuum.

Figure S1 shows the ^{1}H NMR spectrum (performed on a Bruker AM400 NMR spectrometer with deuterium oxide (D_2O) as solvents at 293 K. The chemical shifts were referenced to residual peaks of D_2O at 4.79 ppm) of CM-β-CD in D_2O as well as the assignments. The peaks at 5.32 ppm and 5.10 ppm are assigned to H1; the peaks between 4.50 ppm to 3.30 ppm are contributed by the H2-H6 of CD, the protons of carboxymethyl groups (-CH_2-COOH) and methylene of the residual ethanol (CH_3-C$\boldsymbol{H_2}$OH); and the peak at 1.30 ppm come from methyl of the residual ethanol(CH_3-CH_2OH). The substitution number (SN) of carboxymethyl groups are calculated according to the follow equation [2]:

$$SN = (Area_{4.5\text{-}3.3ppm} - Area_{H2+H3+H4+H5+H6} - Area_{methylene\ of\ ethanol})/2 = (54.36 - 42 - 0.49 \times 2/3)/2 = 6.02 \approx 6$$

Where, the Area 4.5–3.3 ppm denotes the integral from 4.5 to 3.3 ppm; the Area H2 + H3 + H4 + H5 + H6 denotes the summation of integrals of the peaks of H2, H3, H4, H5, and H6, which is 42 because the area of H1 is designated as 7; and the Areamethylene of ethanol is integral of the methylene peak of residual ethanol, which is 2/3 the area of the integral of the peak at 1.30 ppm (methyl of ethanol).

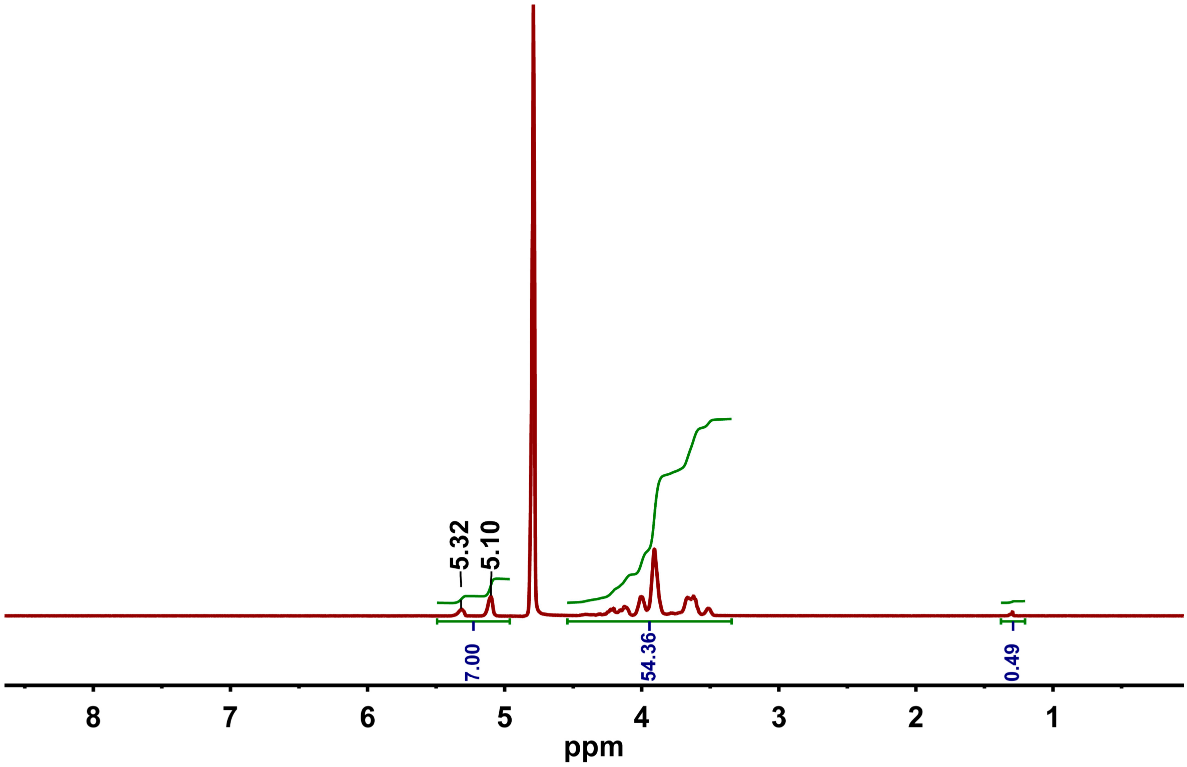

Figure S1. ^{1}H NMR of CM-β-CD.

2. Confirmation of the Supramolecular Interaction Between the CM-β-CD and RBs

To investigate whether the host-guest complexes between the CM-β-CD and RBs were formed, a 2D-NOESY experiment was performed. The concentrations of CM-β-CD, RBs, and $CaCl_2$ in deuterium oxide were 8.4×10^{-4} M, 6.7×10^{-4} M, and 0.04 M, respectively, which were the same as those for preparing CPNPs. As shown in Figure S2A, the cross peaks in red and blue squares clearly show the intermolecular correlations between internal H3, H5 protons of β-CD groups, and the methyl protons of diethylamino groups as well as the aromatic protons of diethylaminophenyl in RB [2]. In addition, it can be seen that there is nearly no signal at the cross section (green square) between the H3 and H5 protons of the β-CD groups and the aromatic protons in RB. The results indicate that complexes of RBs and CM-β-CD formed with the manner that diethylaminophenyl groups of RBs were accommodated into the cavities of CM-β-CD (Figure S2B).

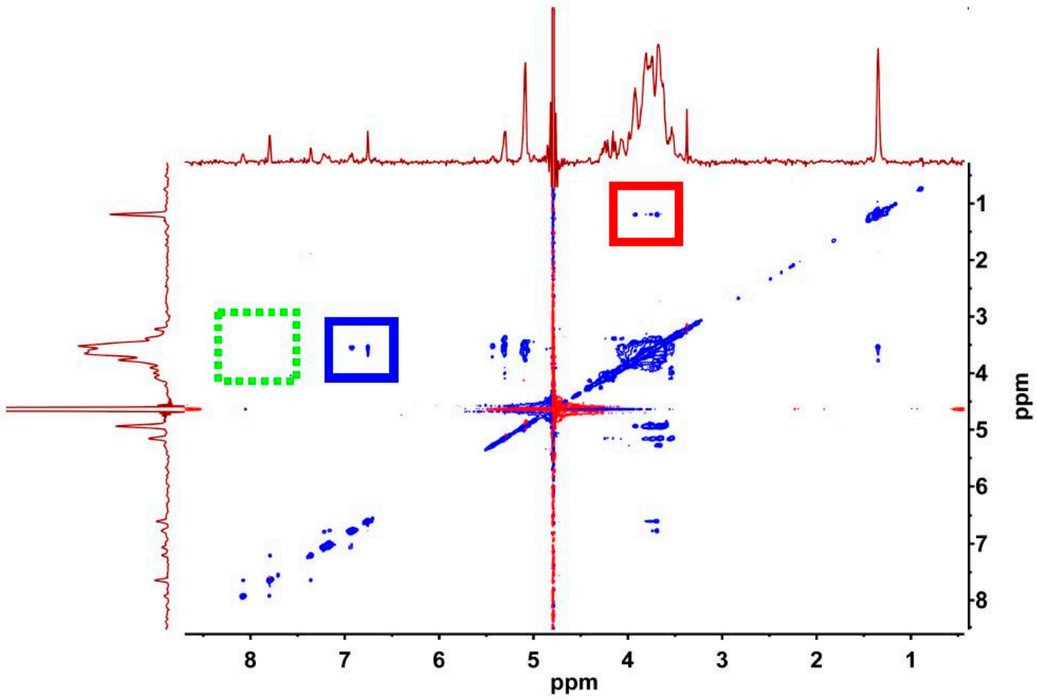

(**A**)

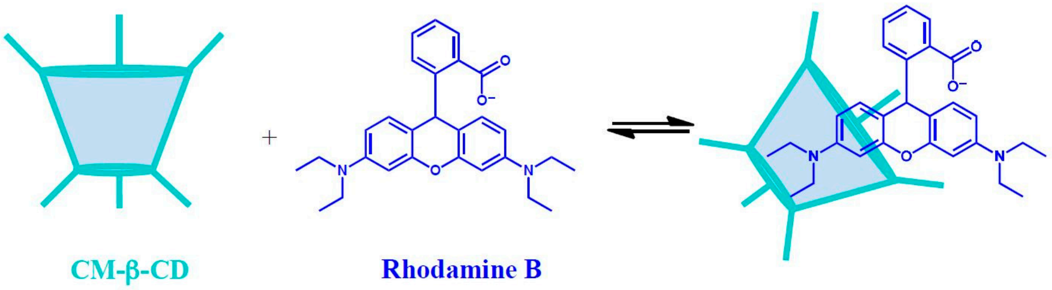

(**B**)

Figure S2. (**A**) 2D NOESY spectrum of CM-β-CD (8.4 × 10^{-4} M) and RB (6.7 × 10^{-4} M) in $CaCl_2$ solution (0.04 M) of D_2O and (**B**) the illustration of the interaction mode between CM-β-CD and RB.

3. The Influence of CM-β-CD to the UV-Vis Absorbance of RBs in PBS

In this work, quantification of RBs was performed in PBS with the presence of CM-β-CD. To evaluate the influence of CM-β-CD to the UV-Vis absorbance of RBs in the PBS conditions, we compared the spectra of RBs in PBS with or without the presence of CM-β-CD. As shown in Figure S3, there are no differences between them, not only in PBS with pH 7.4 (Figure S3A) but also in PBS with pH 5.4 (Figure S3B). The results indicate quantifying RB by UV-Vis absorbance experiments in PBS with the presence of CM-β-CD was accurate.

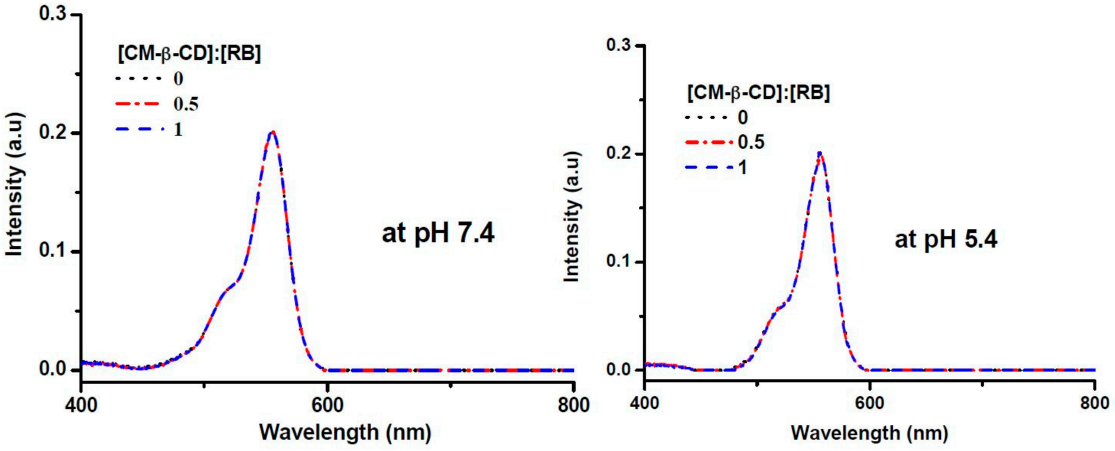

(**A**) (**B**)

Figure S3. UV-Vis absorbance spectra of RB with the addition of CM-β-CD in PBS solution at pH (**A**) 7.4 and (**B**) 5.4.

4. Confirmation of the Supramolecular Interaction between the CM-β-CD and Dtxl

To verify that supramolecular complexes were formed between Dtxls and CM-b-CD, a 2D-NOESY experiment was performed. The concentrations of CM-β-CD, Dtxls, and $CaCl_2$ in deuterium oxide were 1.5×10^{-4} M, 8.4×10^{-4} M, and 0.04 M, respectively, which were the same as for preparing CPNPs. As shown in Figure S4, the signals in the cross section (blue square) clearly reveal the spatial correlations between the methyl groups of Dtxl and H3, H5 of CM-β-CD [4], indicating the formation of a supramolecular complex between Dtxl and CM-β-CD under the condition for preparing CPNPs.

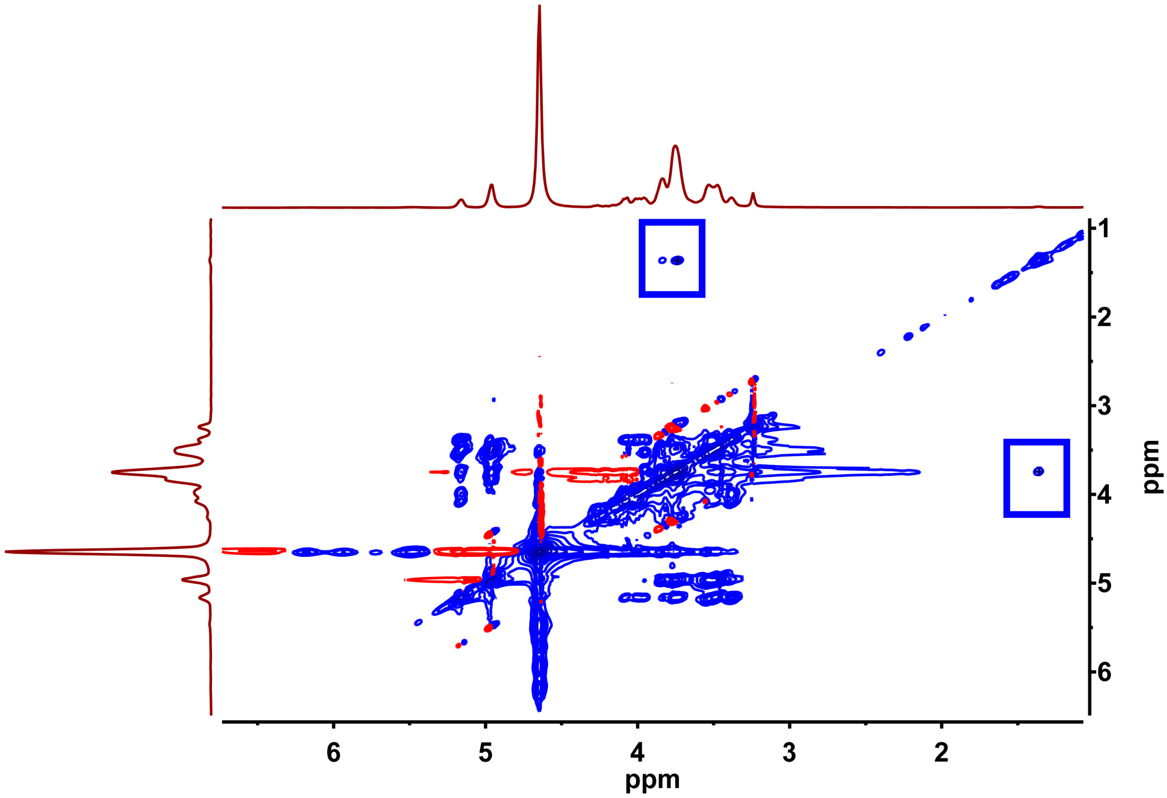

Figure S4. 2D NOESY spectrum of CM-β-CD (1.5×10^{-4} M) and Dtxl (8.4×10^{-4} M) in $CaCl_2$ solution (0.04 M) in D_2O.

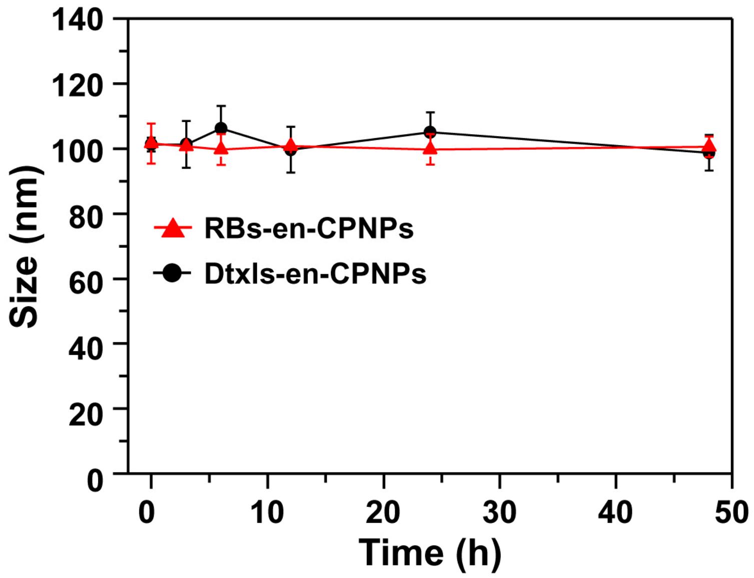

Figure S5. The size change of RBs-en-CPNPs Dtxls-en-CPNPs following incubation with PBS containing 10% FBS.

References

1. Zhi, J.G.; Tian,X. L.; Zhao, W.; Shen, J.B.; Tong, B.; Dong, Y. P. Self-assembled film based on carboxymethyl-β-cyclodextrin and diazoresin and its binding properties for methylene blue. *J. Colloid Interface Sci*. **2008**, 319, 270–276.
2. Wang, X.; Gao, W.; Xu, W.; Xu, S. Fluorescent Ag nanoclusters templated by carboxymethyl-β-cyclodextrin (CM-β-CD) and their in vitro antimicrobial activity. *Mater. Sci. Eng.* **2013**, *33*, 656–662.
3. Liu, Y.; Chen, Y.; S. X. Liu, X. D. Guan, T. Wada, Y. Inoue, Unique fluorescence behavior of rhodamine b upon inclusion complexation with novel bis(β-cyclodextrin-6-yl) 2,2′-bipyridine-4,4′-dicarboxylate. Org. Lett. **2001**, *3*, 1657–1660.
4. Mazzaferroa, S.; Bouchemala, K.; Gallardb, J.F.; Iorgab, B.I.; Cherona, M.; Gueutina, C.; Steinmesse, C.; Ponchel, G. Bivalent sequential binding of docetaxel to methyl-β-cyclodextrin. *Int. J. Pharm.* **2011**, *416*, 171–180.
